# Reward abundance interferes with error-based learning in a visuomotor adaptation task

**DOI:** 10.1371/journal.pone.0193002

**Published:** 2018-03-07

**Authors:** Katinka van der Kooij, Leonie Oostwoud Wijdenes, Tessa Rigterink, Krista E. Overvliet, Joeren B. J. Smeets

**Affiliations:** 1 Vrije Universiteit Amsterdam, Department of Human Movement Sciences, Amsterdam, The Netherlands; 2 Radboud University, Donders Institute for Brain, Cognition and Behaviour, Nijmegen, The Netherlands; 3 Universität Hamburg, Department of Biological Psychology and Neuropsychology, Hamburg, Germany; Centre de neuroscience cognitive, FRANCE

## Abstract

The brain rapidly adapts reaching movements to changing circumstances by using visual feedback about errors. Providing reward in addition to error feedback facilitates the adaptation but the underlying mechanism is unknown. Here, we investigate whether the proportion of trials rewarded (the ‘reward abundance’) influences how much participants adapt to their errors. We used a 3D multi-target pointing task in which reward alone is insufficient for motor adaptation. Participants (N = 423) performed the pointing task with feedback based on a shifted hand-position. On a proportion of trials we gave them rewarding feedback that their hand hit the target. Half of the participants only received this reward feedback. The other half also received feedback about endpoint errors. In different groups, we varied the proportion of trials that was rewarded. As expected, participants who received feedback about their errors did adapt, but participants who only received reward-feedback did not. Critically, participants who received abundant rewards adapted less to their errors than participants who received less reward. Thus, reward abundance negatively influences how much participants learn from their errors. Probably participants used a mechanism that relied more on the reward feedback when the reward was abundant. Because participants could not adapt to the reward, this interfered with adaptation to errors.

## Introduction

Anne’s first beach ball game of the holiday was a frustrating experience: somehow all her balls ended far right of Andy where he could not reach for them. When there are consistent errors in the movements, the brain needs to ‘adapt’ to these biases. This process of bias reduction has been widely studied in visuomotor adaptation paradigms in which participants are exposed to a (rotational) perturbation of visual feedback about their movement. These studies have shown that several processes contribute to the adaptation: use-dependent plasticity, implicit adaptation to ‘sensory prediction errors’, reward-based reinforcement learning and explicit updating of aiming strategy to name a few (for reviews see [1–3]). Here we focus on the combination of two classes of information involved in adaptation: error and reward.

The difference between the planned trajectory of the ball and the actual trajectory provides you with the error you made. This error contains information about the direction in which a correction should be made in the next movement. Noticing that the ball travelled the planned trajectory provides a reward. The absence or presence of a reward provides information on whether or not a correction is needed, but not about the *direction* of this correction. As error and reward provide different types of information, they are held to drive different mechanisms of adaptation [4, 5]. Errors drive mechanisms that rely on correcting the next movement plan with a fraction of the error (e.g. [6, 7–10]). We call this fraction the ‘error sensitivity.’ Reward-based or ‘reinforcement’ learning on the other hand, relies on a combination of exploration and biasing future movement towards rewarded movements [4, 11, 12].

Recent work shows that adding reward to error enhances the overall adaptation. Providing explicit information about success in the form of score rewards can change the speed of adaptation [13–15] and can also improve the retention of adaptation [16–18]. Two explanations have been given for these findings. First, motivational feedback may enhance the sensitivity to error, motivating you to learn more from your errors [13, 19]. Second, reward-based learning may add to error-based learning [4, 13, 19].

In a previous study we found that 3D motor adaptation to endpoint errors was not influenced by binary reward. We attributed this to the fact that the reward in isolation was insufficient for motor adaptation [20], and proposed that reports of enhanced adaptation are due to an addition of error-based and reward-based learning. Alternatively, the influence of reward on motor adaptation could depend on the properties of the reward rule. For instance the proportion of trials rewarded (‘the reward abundance’) may have led to a low motivational value of the rewards such that no measurable influence was found. Here, we test whether the abundance of binary rewards influences motor adaptation.

Participants performed a 3D pointing task that does not induce reward-based adaptation [20]. They pointed to different targets while performance feedback was based on a leftward shifted hand position. Participants were randomly assigned to groups that received positive binary reward feedback for different proportions of trials (low, medium, high). In addition, we compared groups that only received reward feedback (‘reward only’ condition) to groups that received a combination of error and reward feedback (‘reward + error’ condition). To check whether motivation is influenced by reward abundance, we measured motivation immediately after the experimental tasks. If error-based and reward-based adaptation are additive, we expect no influence of reward abundance in the reward + error condition. If reward abundance influences how much participants learn from their errors, we expect an interaction between reward abundance and feedback (reward only, reward+error).

## Methods

### Participants

The study took place in science center Nemo in Amsterdam (www.nemosciencemuseum.nl) as part of their Science Live program (www.sciencelive.nl). Participants were visitors who volunteered to take part, reported to be healthy and were aged between 8 and 65 years. To avoid excluding museum visitors from a museum experience, exclusion criteria that were not related to safety (eye anomaly > 2 diopters, stereo acuity > 100 arcsec, not following the instructions) were applied post-participation but before data analysis. Participants who normally wear contact lenses performed the experiment with their contact lenses, whereas participants who normally wear glasses performed the experiment without their glasses. The study was approved by the Ethics committee of the Faculty of Behavioural and Movement Sciences of the Vrije Universiteit in Amsterdam and all participants provided written informed consent before participating in the experiment. For participants younger than 12 years, a parent signed, for participants 12–16 years old both the child and the parent signed and participants of 16 years or older signed for themselves. Of the 538 initial participants 423 were included in the data analysis (mean age 20.46, SD 14.25; 39 left-handed, 384 right-handed; 193 female, 230 male; 380 Dutch-speaking, 43 non-Dutch speaking). For participants who did not follow the instructions, for instance because they walked around to ‘test’ the VR world, this was noted on their informed consent form. A video impression of the experiment can be viewed online: https://www.youtube.com/watch?v=woRjQ6LeP8U.

### Design

The effect of reward on motor adaptation was assessed in a four (reward) by two (feedback-type) between-participants design. There were two feedback conditions: participants in a ‘reward only’ condition only received reward feedback whereas participants in a ‘reward + error’ condition received a combination of reward and error feedback. There were four reward conditions, three that varied the reward abundance (low, medium, high) and an additional ‘random’ group for whom trials were rewarded at random. Participants were assigned to the eight groups in a pseudo-random order such that different ages were equally represented in the different groups.

### Materials

**Fig 1:**
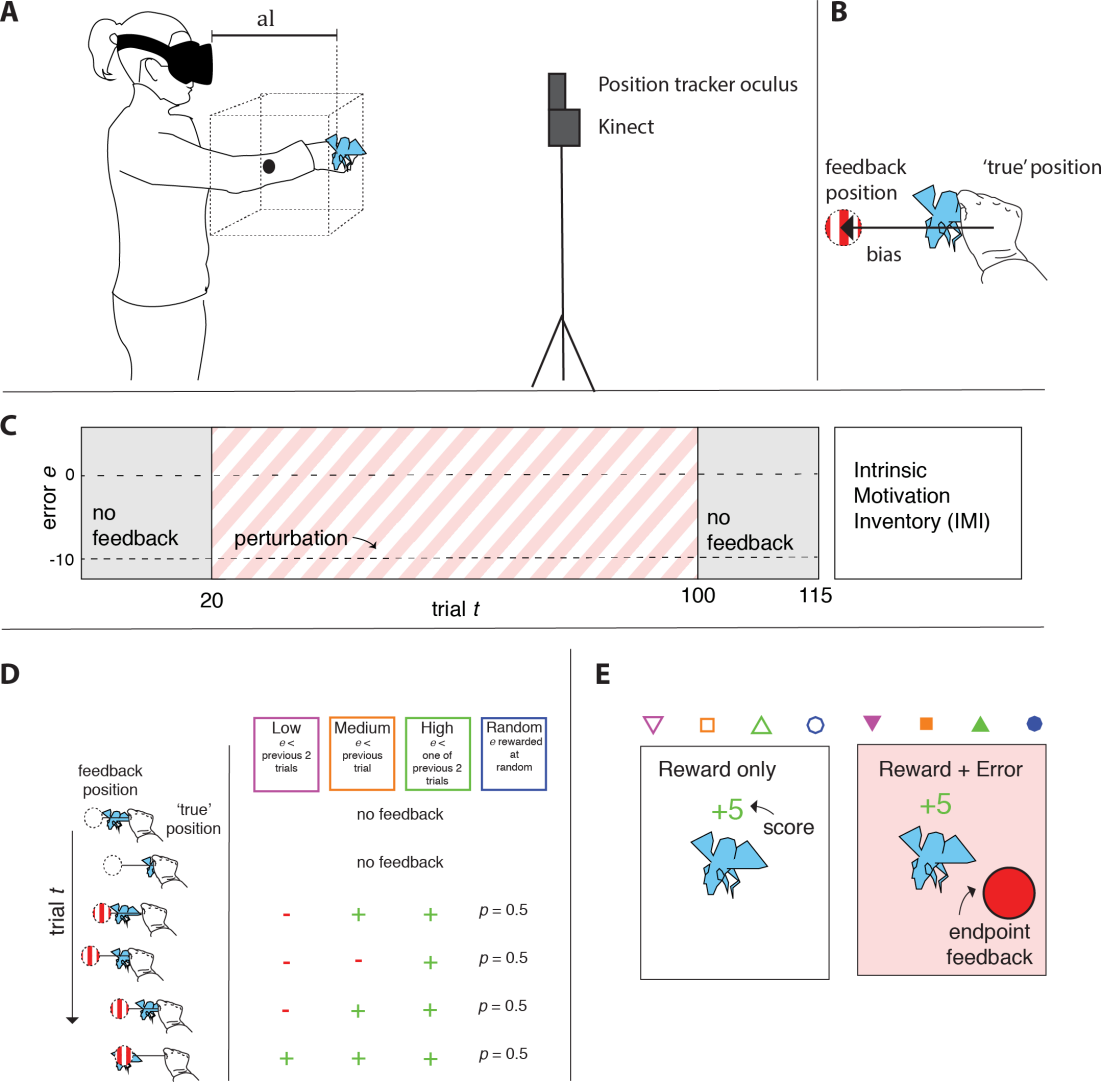
**A)** Experimental set-up showing how the trial area was drawn based on participants’ arm length. **B)** Cartoon illustration of the 10 cm leftward perturbation of the position on which the feedback was based. The blue fly was the movement target. **C**) Procedure, the dotted line indicates the lateral perturbation. **D**) Cartoon illustration of how the reward depended on the error in the different reward conditions. **E**) Reward only and reward + error feedback conditions.

We used a Microsoft Kinect for movement registration, an Oculus Rift DK2 for visual display (resolution 1080 by 1200 for each eye, refresh rate 90 Hz) and Unity 3D for software programming. The Kinect sensor (Fig 1A) was placed onto a tripod at a height of 1.50 meters above the floor, at a distance of approximately 2 meters from the participant and sampled the movement of the dominant hand at a rate of 30 Hz (field of view of 43 by 57 degrees). The Oculus Rift was personalized by setting the player height and interpupillary-distance in the Oculus Rift application. The Oculus positional tracker was attached onto the Kinect such that the lenses of the position tracker and of the Kinect sensor were vertically aligned. To be able to render feedback about the hand position from a first person perspective, we calibrated the coordinate systems of the Oculus Rift and Kinect position data as described in the procedure. During the experiment the perspective from which the participant viewed the stimuli was updated to the tracked position and orientation of the headset, simulating a stable world.

### Procedure

After participants provided written informed consent, their stereo acuity was measured with the Stereo Fly test and the inter-pupillary-distance was measured with a ruler. Participants in the reward-only condition were told that their task was to ‘catch’ virtual flies by bringing their dominant hand to the position where they saw the flies and holding their hand still waiting until the next fly appeared. Participants in the reward + error condition received the same instruction and in addition were told that they would see a red sphere at the position where they had ended their movement which would help them to catch more flies. After having been instructed, participants performed the pointing task, taking about 8 minutes. Two experimenters tested two participants simultaneously, with the participants standing next to each other separated by a room divider.

The participant was asked to stand on an indicated area at 2 meters distance from the Kinect, and was asked to put on the headset. The pointing task started with a calibration procedure. The participant was instructed to stand still, while facing the Kinect camera and extending the arms such that we could record the position of the Oculus Rift headset with the build-in ‘RecenterPose’ function. Next, the Kinect data were centered on this headset position, and aligned with the Kinect floor plane orientation. As a result the coordinate system was centered on the center of the headset and the area in which targets were presented (trial area) was defined based on arm length (*al*; the depth position of the extended dominant hand; Fig 1A). When visual inspection confirmed that the arms were properly extended, a button was pressed to record *al* and the center of the trial area was positioned relative to the position of the headset at that moment at: 0.7 * *al* in front, 0.6 * *al* below and 0.5 * *al* to the right. The size of the trial area was a cube with legs measuring 0.5 * *al*. To introduce a constant bias that participants had to adapt to, a 10 cm leftward lateral shift was added to the tracked position of the dominant hand (Fig 1B). The participants were not told that the feedback was shifted, although some of the participants in the reward + error condition guessed so during the debriefing of the experiment.

Following a five-second countdown, the first target was presented in the center of the trial area, floating in black space and the participant could initiate a movement to align the dominant hand with the position of the target fly (5 cm diameter 3D model). An endpoint was detected when the total displacement of the hand over a period of 300 ms was no more than two centimeters. Depending on the experimental phase, the detection of an endpoint initiated the display of feedback or the appearance of the next target fly at 15 cm distance from the previous target and in a random direction, but such that the target remained within the trial area. Participants moved from fly to fly, without returning to a central starting position.

In the first 20 trials of the experiment, the *baseline phase*, no performance feedback was provided and the next target appeared as soon as an endpoint had been detected. In the middle 80 trials, the *adaptation phase*, feedback was provided as described below and in the last 15 trials, the *retention phase*, again no feedback was provided (Fig 1C).

Reward feedback in the adaptation phase was provided when the pointing error (*e*), the distance between the shifted hand position and the center of the target fly, was smaller than a certain reward criterion *C*, which was based on the participants’ pointing errors in a way that depended on the experimental condition. For the ‘low’ reward condition, *C* was the smaller of the previous two errors, for the ‘medium’ reward condition *C* was the previous error, and for ‘high’ reward condition *C* was the larger of the two previous errors (Fig 1D). For random reward condition, each trial was rewarded at random (50% probability). If the participant was rewarded, positive feedback was provided by adding 5 scored points to the participant’s cumulative score (displayed above the target, Fig 1E), coloring the score green, playing a hit sound and showing an animation of the fly dying. If the error was larger than the reward criterion, no points were scored and the displayed score turned red. Participants in the ‘reward + error’ condition also received feedback about their movement endpoint: a 2 cm in diameter red sphere (Fig 1E) was presented for 500 ms at the shifted hand position.

After finishing the pointing task, participants received their score on a post-it that unbeknownst to the participant was color-coded for the reward group (low, medium, high or random) that they participated in. The post-it was handed together with an iPad that we used to assess motivation with a modified version of the Intrinsic Motivation Inventory (IMI, [21]). To adhere to time constraints of testing participants in the museum, we selected one item from each relevant scale, and in addition, we asked the participant to rate the extent to which they were willing to do the task again (Table 1). The questions were translated to Dutch for Dutch participants; others completed the items in English. For each item, participants indicated on a 5-point scale the extent to which they agreed with the statement.

**Table 1:** The five items used in our modified Intrinsic Motivation Inventory (IMI).

Enjoyment:	“I enjoyed playing this game”
Self-competence:	“I was good at this game”
Effort / importance:	“I tried my best to score as many points as possible”
Tension:	“I felt nervous while I was playing the game”
Motivation to continue:	“I would like to play this game again”

In addition to the IMI items, participants responded to additional questions regarding the participant’s length, handedness and vision. Participants were able to discuss their scores amongst each other during this period, but only received information on the reward group they participated in after completing the motivation questionnaire. Families were instructed and debriefed in a group.

### Data analysis

Data analysis was performed in MatLab R2015a and SPSS version 22.3 was used for statistics. Trials were assigned as outliers based on the size of the 3D error (*e*), which was the difference between the 3D position of the target and the perturbed 3D position of the hand. Errors with a size larger than 2.5 times the standard deviation at that trial within a feedback and reward group were discarded as outliers. This resulted in the exclusion of 2.3% of the trials. Because the perturbation was applied in the lateral dimension, the data analysis focused on the lateral error *e*_*x*_. An analysis of the amplitude of the 3D error (*e*) is provided in Figure A in S1 File.

The adaptation was measured as the asymptotic error *a*, which was the mean lateral error *e*_*x*_ in the last 20 trials of the adaptation phase. To test whether participants responded to the rewards, the amplitude of the trial-by-trial change in e_x_ following rewarded (*Δ*_*reward*_) and non-rewarded trials (*Δ*_*fail*_) were analyzed. The trial-by-trial change *Δ(t)* was the amplitude of the change in error *e*_*x*_ from trial *t* to trial *t*+1. *Δ*_*reward*_ was the mean *Δ(t)* for the rewarded trials *t* in the adaptation phase, whereas *Δ*_*fail*_ was the mean *Δ(t)* for the non-rewarded trials *t* in the adaptation phase. As it might be that the exploration is not specific to the direction of the perturbation, the 3D trial-by-trial change (the amplitude of the change in error *e* from trial *t* tot trial *t*+1) was also analyzed.

The hypotheses that there is a main effect of feedback (reward only, reward + error) on the adaptation and an interaction of reward and feedback were tested by analyzing the asymptotic error in a 4 x 2 univariate ANOVA’s with reward condition (low, medium, high, random) and feedback condition (reward only, reward+error) as between-participants factors. To test whether trial-by-trial changes were larger following non rewarded trials than following rewarded trials [22] *Δ*_*fail*_ and *Δ*_*reward*_ in the random reward group were compared using a Wilcoxon sign rank test. We based this analysis on the random reward group, because only for this group the rewards and the error-size were unrelated. The influence of reward condition on motivation, finally, was analyzed by performing a Kruskal-Wallis rank sum test with reward group as a between-groups factor on the mean rating on the five items of the motivation inventory.

## Results

**Fig 2:**
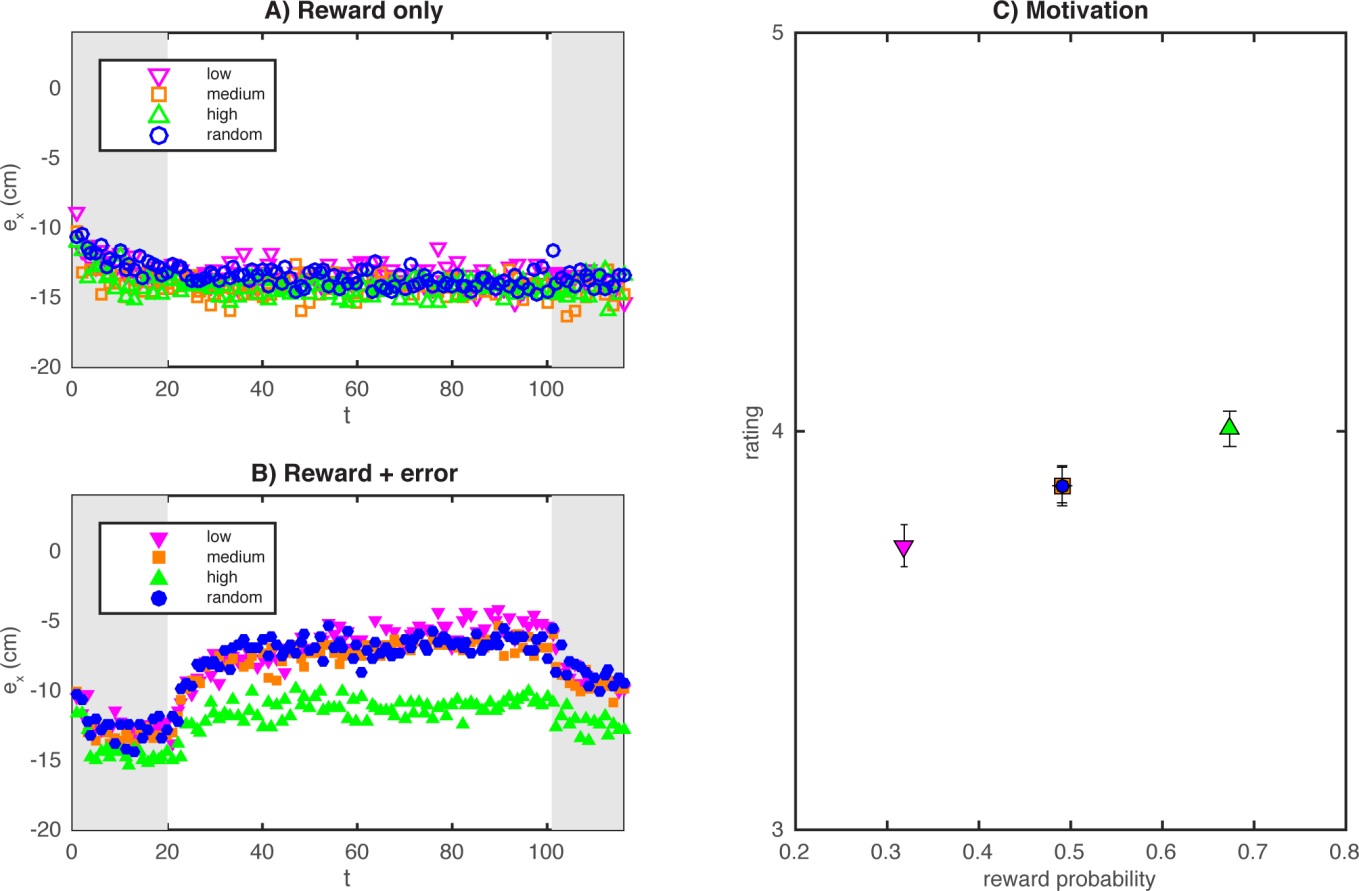
Main results averaged over participants. **A)** Lateral pointing error in the different reward groups of the reward only condition. **B)** Lateral pointing error in the different reward groups of the reward + error condition. **C)** IMI rating for the different reward conditions (low, medium, high, random) as a function of the proportion rewarded trials with standard error of the mean. The results for the random and medium reward condition are overlapping.

Participants in the reward + error condition adapted much more than the participants in the reward only condition (Fig 2A and 2B). The baseline errors start around -10 cm because we calculated the error based on the 10 cm leftward-shifted hand position. Errors appear to drift further leftward during the baseline phase, presumably because the participant’s responses drifted towards a default position (the midline) while the stimuli were consistently presented on the right of the participant.

The ANOVA on the asymptotic error *a* showed a significant main effect of feedback condition (reward only, reward + error) on the asymptotic error (*F*(1,392) = 143.9, *p* < 0.001): on average participants in the reward only condition (Fig 2A) reached an asymptotic error of -12.2 cm whereas on average participants in the reward + error condition reached an asymptotic error of -8.3 cm (Fig 2B). The ANOVA also showed a main effect of reward condition (*F*(3,390) = 5.440, *p* = 0.01) and an interaction of feedback condition and reward condition (*F*(3,390) = 4.046, *p* = 0.007). Post-hoc comparisons with a Bonferroni-corrected *p*-value of 0.008 showed that for the reward only condition (Fig 2A) there were no differences between the reward groups (low, medium, high, random), whereas for the reward + error condition (Fig 2B) the asymptotic error in the high reward group was larger than the asymptotic error in the low reward group (*t*(99) = -4.303, *p* < 0.001), the medium reward group (*t*(99) = -3.190, *p* = 0.002) and the chance reward group (*t*(92) = -3.339, *p* = 0.001). Thus, participants in the high reward group adapted less then participants in the other reward groups. Paired samples t-tests, that compared the asymptotic adaptation in the baseline phase to the asymptotic adaptation in the adaptation phase, showed that participants in all groups that received error feedback adapted: (low reward group: *t*(52) = -8.98, *p* < 0.001; medium reward group: *t*(53) = -8.69, *p* < 0.001; high reward group: *t*(46) = -4.81, *p* < 0.001; random reward group: *t*(46) = -6.92, *p* < 0.001). In Figures B and C in S1 File, we compare the results on the adaptation with model predictions for two possible ways in which reward and error may be combined: ignoring reward errors or weighted combination of error-based and reward-based adaptation.

The high-reward group was on average rewarded in about 68% of the trials, in contrast with 32% for the low-reward group; the other two groups were rewarded in 50% of the trials (horizontal axis Fig 2C). For motivation, the Kruskal-Wallis test showed that it depended significantly on the reward condition (*H*(3) = 16.096, *p* = 0.001). Post hoc Mann-Whitney U tests with a Bonferonni-corrected alpha of 0.008 showed that the motivation in the high reward group was higher compared to the low reward group (*U* = 3569.5, *p* < 0.001). There were no other differences between the other reward groups.

**Fig 3:**
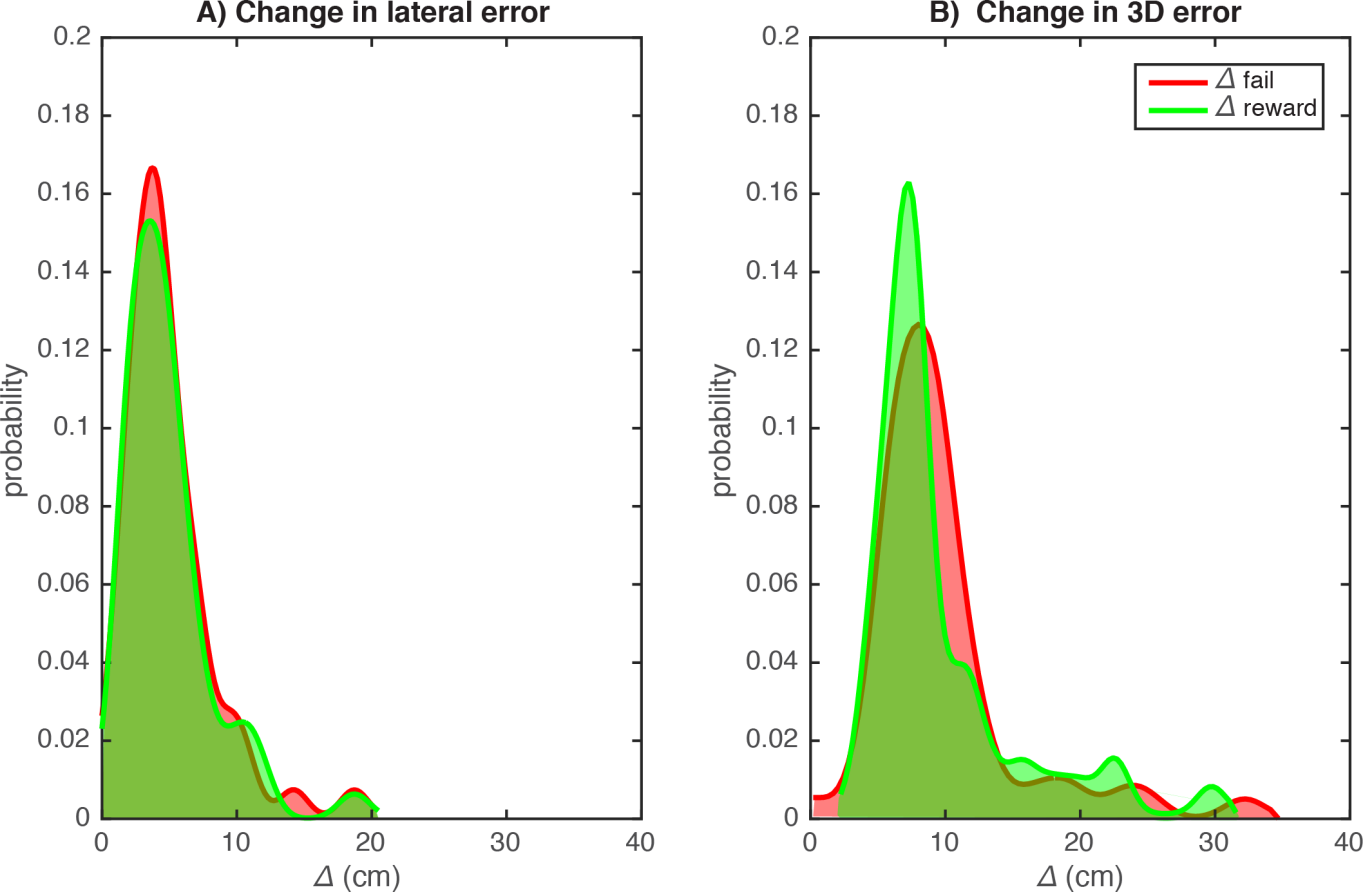
Random reward group, reward only condition. Distribution of trial-by-trial changes in the lateral error following rewarded and non-rewarded trials. **A**) Trial-by-trial changes in the lateral error following randomly rewarded trials (*Δ*_*reward*_) and following randomly non-rewarded trials (*Δ*_*fail*_) show the same distribution. **B**) trial-by-trial changes in the 3D error following rewarded trials were larger than those following non-rewarded trials.

To assess trial-by-trial responses to the reward-feedback, we analyzed trial-by-trial changes in the random reward group of the reward only condition. The mean amplitude of trial-by-trial change in lateral error was 5.1 cm for the rewarded and 5.2 cm for the non-rewarded trials (Fig 3A), which were not significantly different (Wilcoxon signed rank test, *z* = -1.086, *p* = 0.27). In 3D, these changes were obviously larger than for the lateral component: 9.5 cm and 9.9 cm, respectively. Moreover, in contrast with the lateral changes, *Δ*_*fail*_ was significantly larger than *Δ*_*reward*_ for these 3D changes (Wilcoxon signed rank test, *z* = -2.428, *p* = 0.015).

Because a conflict in the information provided by reward and error may influence how they interact, we quantified a possible conflict between the progress indicated by the reward and by the error. We focused on the rewarded trials because reward presence is more informative about the direction of the adaptation than reward absence. In the low reward and medium reward groups there was never a conflict between reward and error. In the high reward group and the random reward group there was sometimes a small conflict in which an error that was larger than the previous error could be rewarded (*R*(*t*) = 1; *e*(*t*) > *e*(*t*-1); high reward group: 20% of trials; random reward group: 25% of trials). Moreover, in the random reward group a larger conflict could occur in which both the reward and error increased relative to the previous trial (13% of trials; *R*(*t*) > *R*(*t*-1) & *e*(*t*) > *e*(*t*-1)). The group that experienced the largest conflict and the highest percentage of conflict trials was the random reward group. As this group did show adaptation, it is unlikely that the reduced adaptation observed for the high reward group is caused by reward and error conflict.

## Discussion

In this study, we tested whether reward abundance influences how much participants adapt to their errors. The results showed that participants in all error feedback groups adapted, but participants in the high reward (68% of the trials) group adapted less to visual errors, compared to when 50% or less of the trials were rewarded (low, medium and random conditions). Although participants in the reward only condition did not adapt, a comparison of trial-by-trial changes following rewarded and non-rewarded trials, showed that 3D changes were larger following non-rewarded trials than following rewarded trials. This indicates that participants did use the reward feedback in their subsequent movement even though they could not learn from it, as has been demonstrated in a previous study [20]). Reports of intrinsic motivation, in contrast to adaptation, depended *positively* on the reward abundance. The influence of reward on motor adaptation and motivation did not depend on the conflict between the reward and error-based feedback: motor adaptation and motivation were equal for a group that received performance-dependent rewards and a group that was rewarded at random (with the same reward abundance).

Our main finding is that participants adapted less to their errors when they received abundant rewards than when they received a smaller amount of reward. Because there was no adaptation when the rewards were presented in isolation, this cannot be explained by the overall adaptation being a sum of error-based and reward-based learning. It has been proposed previously [23] that reward and error are combined on a trial-by-trial basis such that participants only correct for non-rewarded errors. Although this would result in a bit slower adaptation in the abundant reward group, such trial-by-trial combination does not predict the low level of adaptation we observed in this group (see Figure B in S1 File). There are two alternative explanations for the influence of reward on the overall adaptation. Rewards may influence the sensitivity to error or the overall adaptation may be the result of a weighted combination of error-based and reward-based adaptation.

First, it has been proposed that motivational feedback (reward and punishment) enhances the error-sensitivity of error-based adaptation [13, 19], similar to how reward can motivate you to make a faster movement (see [24] for a review). However, we found that the participants who received the most reward adapted the least, yet expressed the most motivation. Although the results on motivation should be interpreted with caution because we used a shortened version of the IMI to accommodate time constraints of testing participants in the museum, the results are difficult to explain by a motivational gain on the error-sensitivity.

The second, and more probable, explanation is that the overall adaptation was based on a combination of error-based processes aimed at reducing error and reward-based processed aimed at scoring points. The finding that trial-by-trial changes in the reward only condition did depend on the reward supports the idea that participants were using the reward feedback in their motor output. Moreover, for rewards that scale with the size of error (‘reward gradient’ or ‘scalar reward’), it has been shown that they contribute to motor learning independently of error-based information and can even induce adaptation in a direction orthogonal to error-based information [25].

One way in which participants could accommodate both error and reward in their adaptation is by simply ignoring errors in rewarded trials [23]. However, this would not explain the reduced adaptation with adbundant rewards (Figure B in S1 File). Another way in which participants could accommodate both error and reward in their adaptation is by taking a weighted sum of error-based and reward-based learning [4]. According to this reasoning, reduced adaptation with abundant rewards would be the result of increased reliance on the reward feedback, which produced no adaptation. In other words, participants in the high reward group focused on scoring points rather than on reducing error (Figure C in S1 File).

Why would participants have relied more on the reward feedback when reward was abundant? Increased reliance on reward-feedback was not due to the conflict between reward and error. The conflict between reward and error was biggest in the random reward group. Yet, participants in the random reward group adapted more than participants in the high reward group. Instead, the reward abundance in the high reward group may have reached a threshold that caused participants to rely more heavily on the reward-based information. Participants viewed a scoreboard before starting the task and knew how many points they should aim for to score above average. The high reward group automatically scored in the higher range and many may have settled for this performance whereas they did not settle for the mediocre performance imposed by the random reward group.

As we noted in the introduction, implicit as well as explicit processes contribute to the adaptation. Implicit processes adapt the movement plan without the participant making any conscious changes in his/her aiming, whereas explicit processes involve changes in an aiming strategy [26–28]. In the current study, probably mainly explicit strategies were involved. We provided terminal feedback, which has been associated with more explicit adaptation [29] and has been shown insufficient for implicit visuomotor adaptation [30]. In addition, we used a relatively large 10-cm rightward perturbation, which was spontaneously reported by a number of participants. In such an explicit process, the absence of reward-based learning may have been mainly due to the spatial complexity of pointing to a different 3D target on each trial rather than to the absence of movement repetition which has been held crucial for implicit reward-based learning [4, 31].

## Conclusion

Reward abundance interfered with error-based adaptation. We propose that when rewards were abundant, participants relied more on the reward feedback. Because they could not adapt to the reward feedback this interfered with adaptation to errors.

## Supporting information

S1 File Supporting information. To check whether we could determine how reward influences motor adaptation, we considered two possibilities: (1) reward influences corrections to error on a trial-by-trial basis or (2) reward influences error-based adaptation on a task-basis. We developed a mechanistic model for each hypothesis and show the model predictions such that they can be compared to the data (Fig A and B). In addition, we report an analysis of absolute errors (Fig C).
(PDF)
