# Correction: Reward abundance interferes with error-based learning in a visuomotor adaptation task

**DOI:** 10.1371/journal.pone.0223088

**Published:** 2019-09-23

**Authors:** Katinka van der Kooij, Leonie Oostwoud Wijdenes, Tessa Rigterink, Krista E. Overvliet, Jeroen B. J. Smeets

The last author’s name is spelled incorrectly. The correct name is: Jeroen B. J. Smeets.

In the Funding statement, the last author’s name is spelled incorrectly. The correct name is Jeroen Smeets.
