# Supplementary material for: Reward abundance interferes with error-based learning in a visuomotor adaptation task
Source: PLoS One. 2018 Mar 7;13(3):e0193002. doi: 10.1371/journal.pone.0193002 (PMC5841744; doi:10.1371/journal.pone.0193002)
Supplement: S1 File — To check whether we could determine how reward influences motor adaptation, we considered two possibilities: (1) reward influences corrections to error on a trial-by-trial basis or (2) reward influences error-based adaptation on a task-basis. We developed a mechanistic model for each hypothesis and show the model predictions such that they can be compared to the data (Fig A and B). In addition, we report an analysis of absolute errors (Fig C). (PDF) [file pone.0193002.s001.pdf]

1 Analysis of absolute errors

To check whether the results were specific to the (lateral) errors in the direction of the perturbation, we also analyzed the size of the 3D error, which we term the 'absolute error'. The mean absolute error in the different reward groups of the two feedback conditions is plotted in S1 Figure A. Note that baseline errors are large because we calculated the error based on the 10° leftward-shifted hand position. As can be seen, participants in the reward only condition (S1 Fig A, left panel) did not reduce their errors in response to the feedback whereas participants in the reward + error condition (S1 Fig A, right panel) did reduce their errors in response to the feedback. Moreover, participants in the high reward abundance group reduced their errors less than participants in the other groups.

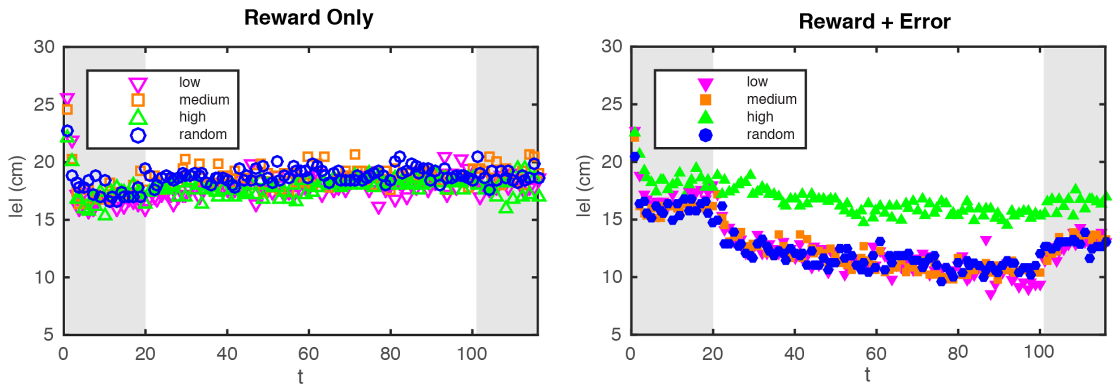

S1 Fig A. Absolute errors averaged over participants. Left panel) The absolute error $|e|$ averaged across participants in the different reward groups of the reward + error condition. **Right panel)** The absolute error $|e|$ in the different reward groups of the reward only condition.

To statistically test whether reward abundance influences 3D adaptation, we analyzed the asymptotic absolute error, which was the mean absolute error in the last 20 trials of the adaptation phase. An ANOVA with feedback condition (reward + error, reward only) and reward condition (low, medium, high, random) as between-participants factors replicated the findings of the ANOVA on the lateral error. There was a main

effect of feedback condition ($F(1{,}392) = 104.133, p < 0.001$). In addition, there was an interaction effect of reward group and feedback condition ($F(3{,}390) = 3.666, p = 0.013$). Post-hoc t-test comparisons with a Bonferonni-corrected alpha of 0.008 showed that for the reward only condition there were no differences between reward groups, whereas for the reward + error condition the asymptotic error in the high reward group was larger (indicating less adaptation) than the asymptotic error in the low reward group ($t(99) = 2.803, p = 0.006$), in the medium reward group (t(99) = 3.680, p < 0.001) and in the random reward group ($t(92) = 3.209, p = 0.002$).

2 How are error and reward combined?

To check whether we could determine how reward influences motor adaptation, we considered two possibilities: reward influences corrections to error on a trial-by-trial basis or reward influences error-based adaptation on a task-basis. We developed a mechanistic model for each hypothesis such that model predictions could be compared to the data.

To analyze the effects of combining error and reward on a trial-by-trial basis, we used a simple state-space estimation model of error-based adaptation. In the model, the lateral error (e) at trial t is determined as the difference between the perturbation Δ and adaptation (X), with added to it motor noise (ζ_m), randomly drawn from a normal distribution with width σ_m. In the model, we assume (Brenner & Smeets, 2011) that participants retain a fraction A from their adaptation in the previous trial, correct their adaptation with a fraction B of the error for non-rewarded trials ($R = 0$), but do not correct for rewarded trials (R=1):

$$e(t) = \Delta - X(t) + \zeta_m \quad (1.1)$$

$$X(t+1) = A\,X(t) - (1 - R)\,B\,e(t) \quad (1.2)$$

To run the model simulations, we chose A and B on an equally spaced 50-element grid from 0 to 1 and ran 1000 model simulations for each combination of A and B. Motor noise σ_m was set to 3 cm such that it roughly matched the standard deviation

observed in the baseline phase of the experiment. Ignoring rewarded errors yields for each combination of *A* and *B* an amount of adaptation that depends on the reward scheme (S1 Fig B). However, the observed differences in adaptation are not following these predictions. To explain the reduced adaptation in the high reward group with this model, rewards should thus affect the learning rate or the retention.

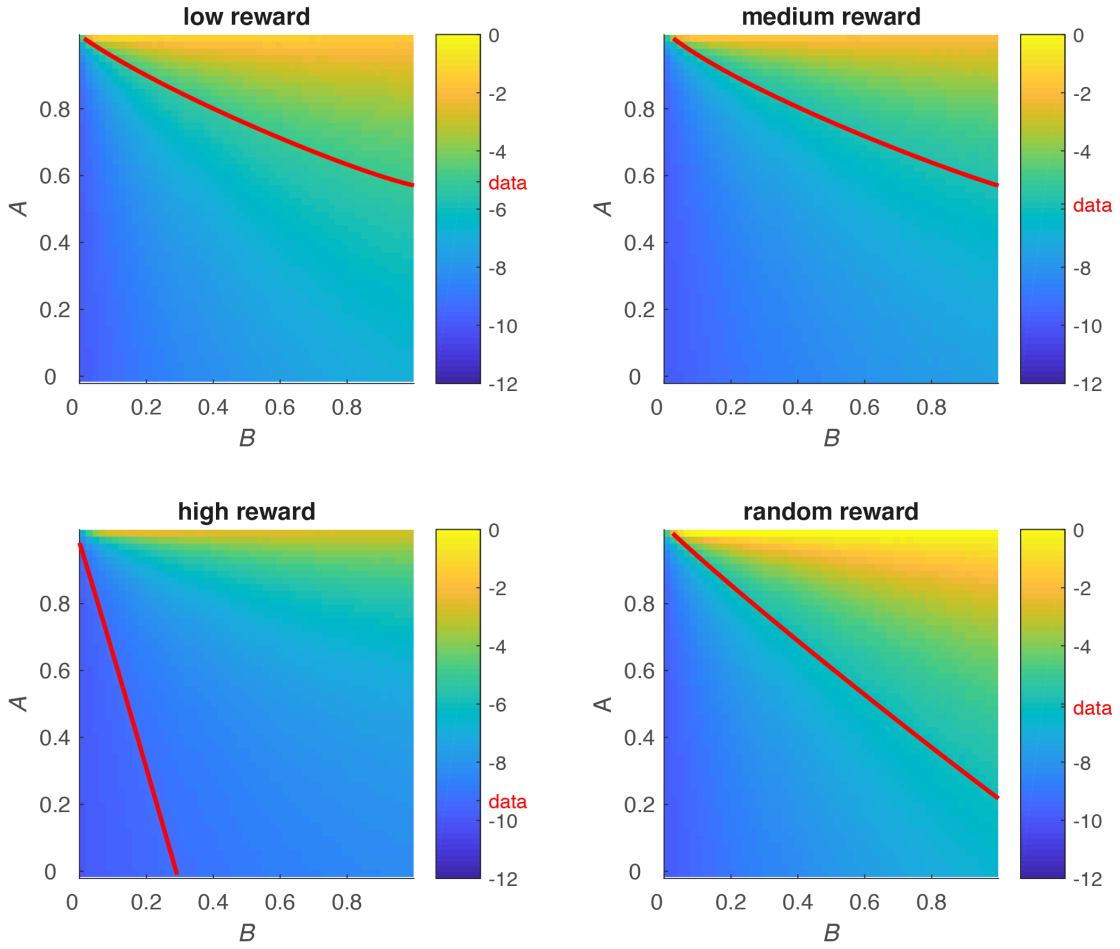

S1 Fig B Influence of ignoring rewarded error on the adaptation*. Heat map of the adaptation predicted by equations (1.1)-(1.2) (averaged over 1000 samples) in the reward+error condition as a function of B and A. Red lines represent values that overlap with the data. The four reward schemes yield a different adaptation for a given set of parameters, but these differences do not correspond to the differences in the observed amount of adaptation.*

To analyze how error and reward would be combined on a task-basis, we combined a simple model of reward-based adaptation with a model of error-based adaptation similar to the one that is also used in the analysis above (Equations (1.1) and (1.2) with R=0). As a model for reward-based adaptation we used a model proposed by Therrien et al. (2016), in which the error e_x on trial *t* is a combination of motor noise ζ_m, exploration ζ_η and adaptation state *X*. Motor noise and exploration are both stochastic variables drawn from a normal distribution with standard deviation σ_m and σ_η respectively. When the trial is rewarded because the error is smaller than reward criterion C (see methods, expressed by the Heaviside function H in the equations), the state is updated with the exploration at that trial, otherwise nothing happens. The total adaptation (X) can now be written as a weighted combination of error-based adaptation (X_E) and reward-based adaptation (X_R):

$$X(t) = (1 - w)\, X_E(t) + w\, X_R(t) \tag{2.1}$$

$$e(t) = \Delta - X(t) + \zeta_m + \zeta_\eta \tag{2.2}$$

$$X_E(t+1) = A\, X_E(t) - B\, e(t) \tag{2.3}$$

$$X_R(t+1) = X_R(t) + H(C - |e(t)|)\, \zeta_\eta \tag{2.4}$$

We ran 1000 model simulations in which we varied *B* and *w* on a 50-element grid ranging from 0 to 1. As we did in the previous analyses, σ_m was set to 3 cm. Exploration noise σ_η was set to 0.5 cm based on exploratory model fits to the data. *A* was set to 0.8. S1 Figure C shows the resulting adaptation averaged over 1000 model simulations. The adaptation barely depended on the reward group. Moreover, different combinations of *w* and *B* resulted in an adaptation that matched the data. Therefore it is difficult to disentangle based on behavioral data which parameter is influenced by the reward condition.

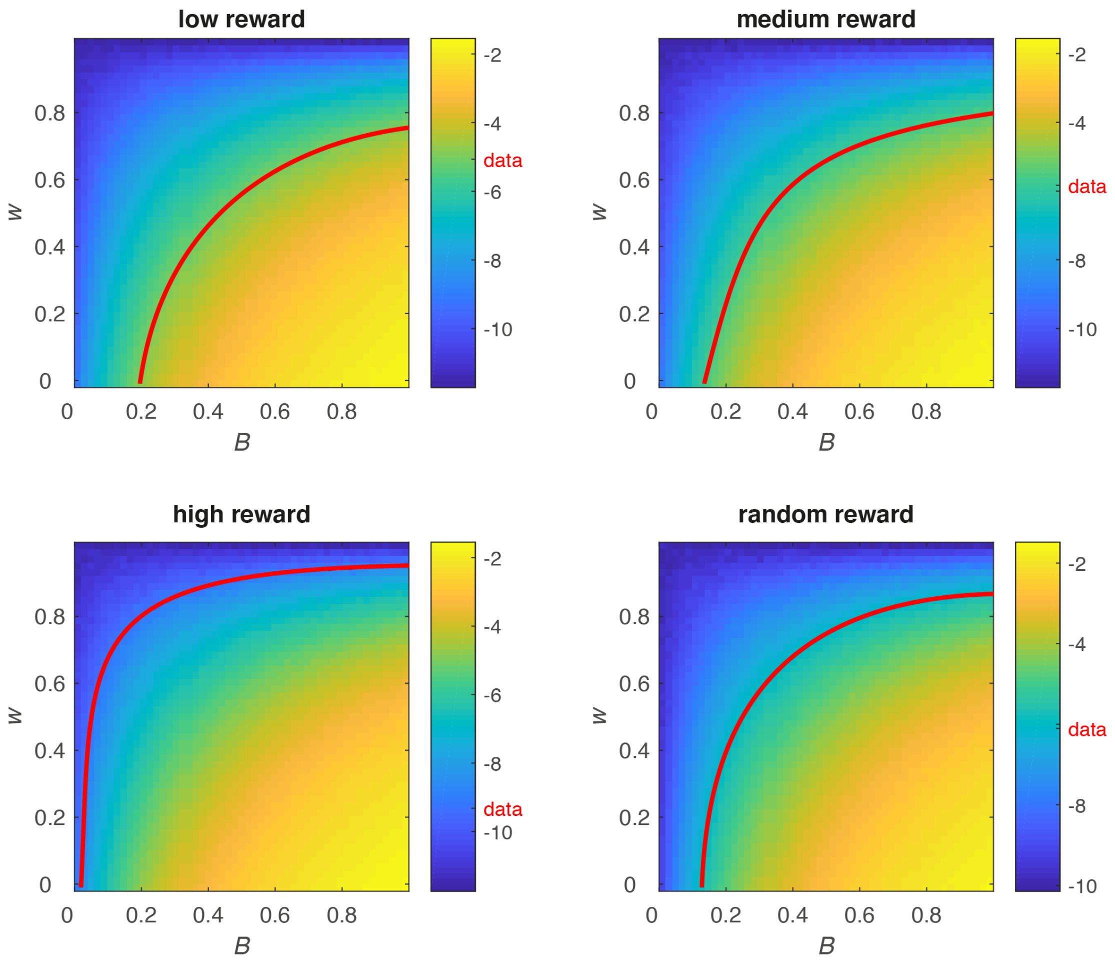

S1 Fig C. The effects of weighting of reward-based information and learning rate on the adaptation*. Heat map of the predicted adaptation (averaged over 1000 samples) in the reward + error condition as a function of B and w. Red lines represent values that overlap with the data. The plot suggests that participants weight reward-based information higher in the high-reward group. However, we cannot exclude that the learning rate B is reduced instead.*

References

Brenner, E., & Smeets, J. B. J. (2011). Quickly 'learning' to move optimally. *Experimental Brain Research, 213*, 153-161.

Therrien, A. S., Wolpert, D. M., & Bastian, A. J. (2016). Effective reinforcement learning following cerebellar damage requires a balance between exploration and motor noise. *Brain, 139*, 101-114. doi:10.1093/brain/awv329
